# 高效液相色谱法测定甲油胶中2，4，6-三甲基苯甲酰基-二苯基氧化膦等5种光引发剂

**DOI:** 10.3724/SP.J.1123.2025.06027

**Published:** 2025-12-08

**Authors:** Xu GONG, Jing SUN, Xinxin LI, Youlong FENG

**Affiliations:** 1.江苏省药品监督检验研究院，江苏 南京 210019; 1. Jiangsu Institute for Drug Control，Nanjing 210019，China; 2.南京中医药大学药学院，江苏 南京 210023; 2. School of Pharmacy，Nanjing University of Traditional Chinese Medicine，Nanjing 210023，China

**Keywords:** 高效液相色谱, 甲油胶, 光引发剂, 2，4，6-三甲基苯甲酰基-二苯基氧化膦, 化妆品, 酰基氧化膦类, high performance liquid chromatography （HPLC）, gel nail polish, photoinitiators （PIs）, 2，4，6-trimethylbenzoyl diphenyl phosphine oxide （TPO）, cosmetics, acylphosphine oxides

## Abstract

甲油胶因快速固化、色泽艳丽、持久耐用等优势成为化妆品消费热点，但其关键组分光引发剂（PIs）存在健康风险，尤其是2，4，6-三甲基苯甲酰基-二苯基氧化膦（TPO）具有生殖毒性已被欧盟全面禁用。目前，该类成分在我国尚存在监管盲区，相关分析方法也较为匮乏。本研究建立了甲油胶中5种酰基氧化膦/*α*-羟基酮类PIs同时定量的分析方法。样品经乙腈超声提取，采用Kromasil 100-5-C18柱（150 mm×4.6 mm，5 μm）进行高效液相色谱分离，以水-乙腈为流动相进行梯度洗脱，检测波长为243 nm和375 nm，采用标准曲线外标法定量。实验结果表明，TPO、2，4，6-三甲基苯甲酰基苯基膦酸乙酯（TPO-L）、2，4，6-三甲基苯甲酰基-二（对甲苯基）氧化膦（TMO）、双（2，4，6-三甲基苯甲酰基）苯基氧化膦（PB-TMBPO）和1-羟基环己烷苯酮（HCHPK）与甲油胶基质干扰峰分离度良好，5种PIs在2~600 mg/L范围内线性关系良好（相关系数*r*均≥0.999 9），方法检出限为3.6~45 μg/g，定量限为15~141 μg/g，在低、中、高3个加标水平下的回收率为91.6%~101.8%，相对标准偏差（RSD，*n*=6）为0.2%~4.3%。将该方法应用于网络购买的30批甲油胶样品的检测，结果所有样品均含1~3种未标识的PIs，总含量达到2.95%~9.66%（质量分数）。结果表明TPO等PIs在当前甲油胶市场上被高频使用，亟需监管、替代和进一步安全评估。该方法操作便捷，分离效率高，专属性强，能够在低成本下满足甲油胶中PIs含量测定的需求，为甲油胶产品的质量控制和安全性评估提供了技术支持。

## Abstract (en)

Gel nail polish has surged in global popularity， becoming a dominant force in the cosmetics market due to its compelling advantages. This rapid market expansion， however， is shadowed by significant and growing safety concerns related to the essential chemical agents enabling its functionality—photoinitiators （PIs）. Classified as a Category 1B CMR substance （carcinogenic， mutagenic， or toxic to reproduction） based on robust evidence of reproductive toxicity， 2，4，6-trimethylbenzoyl diphenyl phosphine oxide （TPO） has been comprehensively banned within the European Union （EU） under Regulation （EC） No 1223/2009， effective from September 1， 2025. Compounding this challenge was the conspicuous absence of validated analytical methods. Specifically， standardized， reliable， and accessible techniques for quantifying the complex spectrum of PIs in modern gel nail polish formulations were critically lacking. This included established PIs such as the now-banned TPO and its potential substitutes， all of which were embedded within the challenging， heterogeneous matrix of these formulations. This methodological gap severely hindered effective quality control during manufacturing， robust post-market surveillance by regulators， accurate consumer exposure assessment， and the essential safety evaluation of emerging replacement PIs. Consequently， there was an immediate and pressing need to develop dedicated analytical capabilities capable of monitoring both prohibited high-risk PIs and their prospective successors within this specific product category. To address the critical absence of standardized analytical methods for PIs in cosmetics， this study developed and validated a robust high performance liquid chromatography with diode array detection （HPLC-DAD） protocol. The protocol enables the simultaneous quantification of five strategically selected PIs in gel nail polish formulations. These PIs include TPO， its structural analogues ethyl （2，4，6-trimethylbenzoyl） phenylphosphinate （TPO-L）， ［bis（4-methylphenyl） phosphinyl］ （2，4，6-trimethylphenyl） methanone （TMO）， and ［phenyl（2，4，6-trimethylbenzoyl） phosphoryl］ （2，4，6-trimethylphenyl） methanone （PB-TMBPO）， as well as the α-hydroxy ketone 1-hydroxycyclohexyl phenyl ketone （HCHPK）. This target panel encompasses both the currently dominant PIs and prime TPO replacement candidates. The optimized sample preparation involved efficient extraction of target analytes from the complex gel nail polish matrix using acetonitrile under ultrasonication， eliminating the need for derivatization. Chromatographic separation was achieved on a Kromasil 100-5-C18 column （150 mm×4.6 mm， 5 μm） maintained at 30 ℃. A binary mobile phase gradient （water/acetonitrile） was delivered at 1.0 mL/min， with an injection volume of 5 μL. Selective detection leveraged dual wavelengths： 243 nm optimized for HCHPK and 375 nm for the four acylphosphine oxides. Quantitation employed external calibration curves. Method validation demonstrated exceptional performance： All five PIs exhibited baseline separation from matrix interferences. Linear calibration curves spanned 2–600 mg/L with outstanding correlation coefficients （*r≥*0.999 9 for each analyte）. Method sensitivity was confirmed with limits of detection （LODs） ranging from 3.6 to 45 μg/g and limits of quantitation （LOQs） from 15 to 141 μg/g. Accuracy， assessed through spiked recovery experiments at low， medium， and high concentration levels， yielded excellent results （91.6%–100.4%）. Precision， expressed as relative standard deviation （RSD， *n=*6）， was consistently high （0.3%–4.3%）， affirming method robustness and reliability for routine analysis. Application of the validated method to 30 commercially available gel nail polish samples revealed pervasive PI presence. Total PI content ranged from 2.95% to 9.66% （mass fraction）. Each batch contained 1 to 3 distinct PIs. None of these PIs were listed on the product labels. Critically， TPO， which has been banned in Europe， was present in 90% of the analyzed products at significant concentrations （1.42%–6.43%， mass fraction）. HCHPK was detected in 76.7% of the samples， with levels ranging from 0.83% to 4.84% （mass fraction）. TPO-L was present in 10% of the samples （6.73%–7.17%， mass fraction）， while TMO was detected at 0.25% （mass fraction） in a single sample. These findings indicate the prevalent use of prohibited substances like TPO and other PIs in the current gel nail polish market. Enhanced regulation， substitution efforts， and further safety assessments are urgently required. This study delivers a simple， specific， rapid， and economically viable HPLC-DAD method specifically tailored for the challenging gel nail polish matrix. It effectively addresses the analytical gap for monitoring key PIs， particularly during the critical transition period following the EU TPO ban. The methodology provides robust technical support for quality control laboratories （both industrial and regulatory）， facilitates compliance verification with evolving safety regulations， enables accurate risk assessment of consumer exposure， and empowers the development of safer gel nail polish formulations through reliable PI quantification. The alarming market data generated underscores the immediate necessity for regulatory action and industry innovation toward eliminating high-risk PIs like TPO.

随着社会经济的发展和人们生活水平的显著提升，化妆品产业在美学需求驱动下迎来高速发展期。甲油胶（gel nail polish）是一类需要借助紫外线（紫外线发光二极管灯）照射才能固化的新型美甲产品。主要产品类型包括底胶、封层胶、色胶等；色胶种类繁多，可细分为纯色胶、亮片胶、渐变胶等^［1］^。其核心成分为基础树脂和光引发剂（photoinitiators，PIs），并辅以颜料、稀释单体、流变改性剂、交联剂等^［1］^，共同构建起兼具高效固化、高色牢度与高可塑性的美妆体系。PIs是光固化过程的关键物质，能够吸收特定波长紫外线，产生活性中间体（自由基或阳离子），进而引发单体和紫外（UV）固化树脂聚合、交联及接枝反应，最终实现甲油胶的快速固化^［2］^。

然而，作为甲油胶光固化反应的“分子开关”，PIs带来的安全风险不容忽视。例如，2，4，6-三甲基苯甲酰基-二苯基氧化膦（TPO）因具有生殖毒性，被欧盟列为推定对人类有生殖毒性（presumed human reproductive toxicant，1B）类致癌性、致突变性或生殖毒性（carcinogenic， mutagenic or reprotoxic，CMR）物质，2023年被列入高关注物质（substances of very high concern candidate list，SVHC）候选清单^［3］^，并已于2025年9月1日起在欧盟全面禁用。这一变化将迫使光固化行业加速寻找并转向更安全的TPO替代品^［4］^，如2，4，6-三甲基苯甲酰基苯基膦酸乙酯（TPO-L）、2，4，6-三甲基苯甲酰基-二（对甲苯基）氧化膦（TMO）和双（2，4，6-三甲基苯甲酰基）苯基氧化膦（商业名Irgacure 819*，*简称PB-TMBPO）。然而，不管是TPO还是其替代物，仍可能存在皮肤致敏性、细胞毒性或内分泌干扰效应等安全隐患。因此，建立准确、灵敏、高效的分析方法以实现甲油胶产品中PIs定性、定量检测，并对其进行严格监控，对于保障消费者健康安全、满足日益严格的法规要求（如欧盟化妆品法规（EC） No 1223/2009）以及指导安全配方的研发，均具有极其重要的意义。

目前我国甲油胶中PIs的检验标准缺失，尚未要求产品标签和备案资料标识该类成分，存在监管和安全风险评价盲区。国内外PIs的检测研究主要集中在紫外固化油墨、3D打印材料及食品接触材料等领域^［5］^，化妆品领域的检测研究匮乏。现有检测方法包括气相色谱-质谱联用法（GC-MS）^［6，7］^、液相色谱-串联质谱法（HPLC-MS/MS）^［8，9］^、液相色谱-紫外检测法（HPLC-UV）^［10］^及超高效合相色谱^［11］^等。GC-MS方法虽文献报道较多，能够通过保留时间和特征碎片离子实现准确可靠的定性分析^［6］^，但对于热不稳定或难挥发的PIs适用性受限；HPLC-MS/MS虽然具有高选择性，但对于难电离的小分子PIs响应不佳，且仪器成本高，方法开发复杂，不适用于常量分析；HPLC-UV操作简便，但在复杂基质下选择性不足，容易受到干扰；高效合相色谱以CO₂为主要流动相，虽绿色、环保，但其仪器普及难度较大。

鉴于甲油胶成分复杂、目标物含量未知且种类多样，尤其在新旧PIs更替期，亟须一种兼具良好选择性、灵敏度、普适性及高效性的检测方法。本研究基于HPLC结合二极管阵列检测器（DAD）在常规实验室中的高普及率和较低运行成本，开发并验证一种高效、可靠、适用于常规实验室的HPLC-DAD检测方法，用于同时测定甲油胶中5种关键PIs（结构式见图1）：TPO、TPO-L、TMO、PB-TMBPO和1-羟基环己烷苯酮（商业名Irgacure 184，简称HCHPK）。研究通过系统优化样品前处理流程和色谱分析条件，排除复杂基质干扰，提高分离效率和检测灵敏度，为甲油胶产品质量安全控制、PIs使用合规性评估及风险监测提供了一种简便、实用、高效的分析手段。

**图1:**
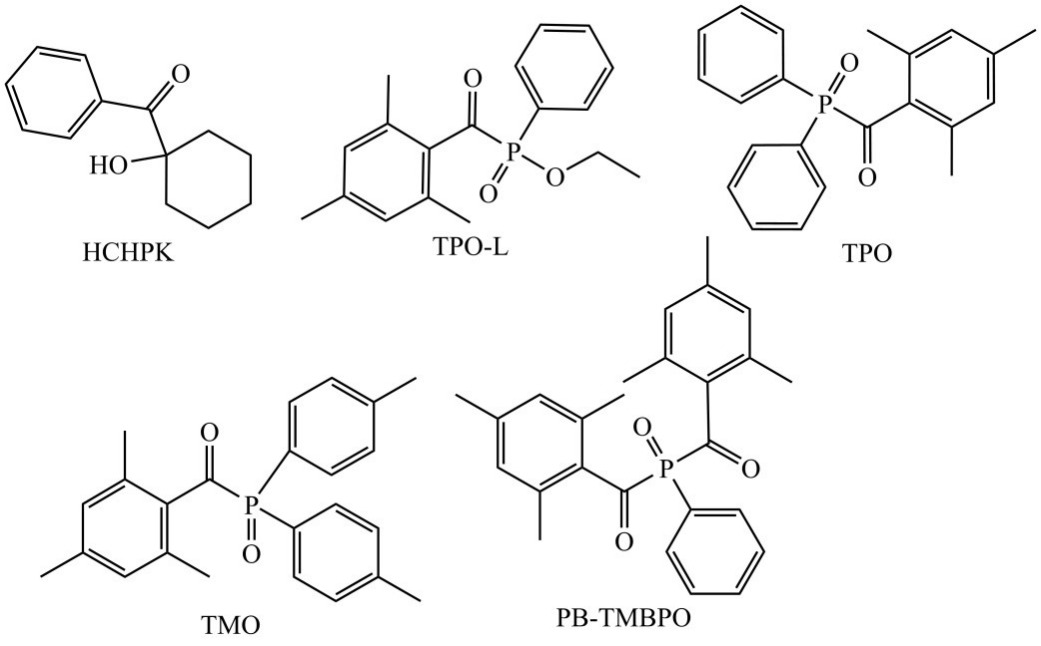
5种光引发剂的结构式

## 1 实验部分

### 1.1 仪器、试剂与材料

LC-30AD超高压液相色谱仪（日本岛津公司），配有SIL-30AC自动进样器、CTO-20AC色谱柱恒温箱、SPD-M20A二极管阵列检测器（检测波长为190~800 nm）、LabSolutions色谱工作站；MSE224S万分之一分析天平（德国Sartorius公司）；XSR205DU十万分之一分析天平（美国Mettler-Toledo公司）；XP6百万分之一分析天平（美国Mettler-Toledo公司）；UA22MFD超声仪（德国Wiggens公司）。

HCHPK（C_13_H_16_O_2_，CAS号947-19-3，纯度99.6%）、TPO（C_22_H_21_O_2_P，CAS号75980-60-8，纯度98.6%）、PB-TMBPO（C_26_H_27_O_3_P，CAS号162881-26-7，纯度98.6%）购自上海安谱璀世标准技术服务有限公司；TPO-L（C_18_H_21_O_3_P，CAS号84434-11-7，纯度97.0%）、TMO（C_24_H_25_O_2_P，CAS号270586-78-2，纯度98.0%）购自上海麦克林生化科技股份有限公司；甲醇、乙醇、乙腈，均为色谱纯，购自德国Merck公司；甲酸为色谱纯，购自美国ACS恩科化学公司；丙酮为色谱纯，购自国药集团化学试剂有限公司；试验用水为超纯水，由Milli-Q IQ 7000超纯水系统制得。30批次甲油胶样品均购自中国电商平台淘宝网，涵盖4个品牌畅销产品，包含底胶（MC1、G10）、封层胶（MC2~7、G8~9）、流平胶（MC8）、纯色胶（MC9~11、MN4~6）、亮片胶（G1~7、MN1~3）和渐变胶（YH1~3）等功能类型。样品采购时间集中于2025年5~6月，剂型囊括液体、黏稠液体和凝胶。

### 1.2 标准溶液的配制

分别称取HCHPK、TPO-L、TPO、TMO和PB-TMBPO各100 mg，置于同一50 mL棕色量瓶中，用乙腈溶解并定容至刻度，配制成5种分析物质量浓度均为2 mg/mL的混合标准储备液，-20 ℃下密封保存。量取适量混合标准储备液，用乙腈逐级稀释成质量浓度分别为600、400、200、100、40、10、4、2 mg/L的系列混合标准溶液。

### 1.3 样品前处理

样品混匀，称取0.1 g（精确到0.000 1 g），置于10 mL棕色具塞比色管中，加乙腈定容至刻度，密塞涡旋1 min，使试样分散于提取溶剂中，再超声提取20 min（功率500 W、频率40 kHz），过0.45 μm有机滤膜，取续滤液供HPLC测定。若目标成分检出浓度超出线性范围，则用乙腈按一定比例进行稀释后测定。

### 1.4 色谱条件

色谱柱：Kromasil 100-5-C18柱（150 mm×4.6 mm，5 μm）；柱温：30 ℃；流动相：A为水，B为乙腈；梯度洗脱程序：0~2 min，55%B；2~6 min，55%B~95%B**；**6~8 min，95%B；8~8.5 min，95%B~55%B**；**8.5~12 min，55%B。流速：1 mL/min；进样体积：5 μL；HCHPK的检测波长为243 nm，TPO-L、TPO、TMO和PB-TMBPO的检测波长为375 nm。

## 2 结果与讨论

### 2.1 色谱条件的优化

#### 2.1.1 检测波长的选择

甲油胶样品大量干扰峰的存在严重阻碍了目标化合物的精准检测，为有效应对这一难题，本研究采用二极管阵列检测器，在190~800 nm波长范围内对5种PIs进行光谱扫描，结果显示，HCHPK在243 nm处有较强的吸收，而TPO-L、TPO、TMO和PB-TMBPO在236、296、375 nm处有较强吸收。然而，甲油胶样品中的干扰物质在≤300 nm波长范围内存在不同程度的吸收，可能会对TPO-L、TPO、TMO和PB-TMBPO的检测产生干扰。为兼顾检测灵敏度与专属性，本研究采用双波长检测策略：以243 nm作为HCHPK的检测波长，375 nm作为TPO-L、TPO、TMO和PB-TMBPO的检测波长。

#### 2.1.2 流动相的选择

为优化色谱分离条件，系统考察了不同流动相组合对5种PIs色谱行为的影响。实验分别采用水和0.1%（体积分数）甲酸水溶液作为流动相A，以及甲醇、乙腈、甲醇-乙腈（1∶1，体积比）作为流动相B。结果显示，在相同梯度洗脱条件下，尽管两种水相的使用对5种PIs的保留时间和峰形的影响不大，但在243 nm波长下0.1%甲酸水溶液存在末端吸收，导致基线随梯度变化而波动，影响检测稳定性。因此，本研究选择水作为水相。在有机相的选择上，鉴于酰基氧化膦类PIs色谱保留较强，以甲醇作为有机相时，仪器分析时间较长且峰形较宽，难以满足快速、高效分离的需求。而乙腈能在保证各PIs良好峰形和分离度的同时，更快出峰，显著缩短仪器分析时间。基于以上结果，本研究最终选择水-乙腈体系作为流动相，梯度洗脱。

#### 2.1.3 色谱柱的选择

色谱柱的选择对于实现5种目标PIs与复杂样品基质中干扰物的有效分离至关重要。尽管选择375 nm检测波长，使TPO等4种酰基氧化膦类PIs受到甲油胶基质的干扰较少，分离效果较为理想，但在243 nm检测波长下定量HCHPK时，甲油胶基质的干扰峰较多，对色谱分离提出了极高要求。因此，实验选用多种色谱柱进行比较。所考察的色谱柱包括Kromasil 100-5-C18（150 mm×4.6 mm，5 μm）、Venusil MP C18（150 mm×4.6 mm，5 μm）、Diamonsil plus C18（150 mm×4.6 mm，5 μm）和Poroshell 120 EC-C18（100 mm×4.6 mm，4 μm）。考察所用样品为辅料干扰严重的一批样品（MC2），该样品含有TPO（峰号为3），各色谱柱其分离效果如图2所示。向MC2样品溶液中加入另外4种未检出的PIs，计算5种PIs在各色谱柱上的理论板数如表1所示。结果表明，在所考察的色谱柱中，Kromasil 100-5-C18柱对目标峰和干扰峰均展现出卓越的峰形表现，平均理论塔板数最大，并且在梯度洗脱时间内实现了5种PIs与样品中干扰物的最佳分离。尤其在243 nm波长下，HCHPK与样品中干扰峰的分离度（*R*）>3，有效避免了甲油胶基质的干扰，从而确保了HCHPK定量的准确性和可靠性。因此本实验选择Kromasil 100-5-C18（150 mm×4.6 mm，5 μm）色谱柱。

**图2:**
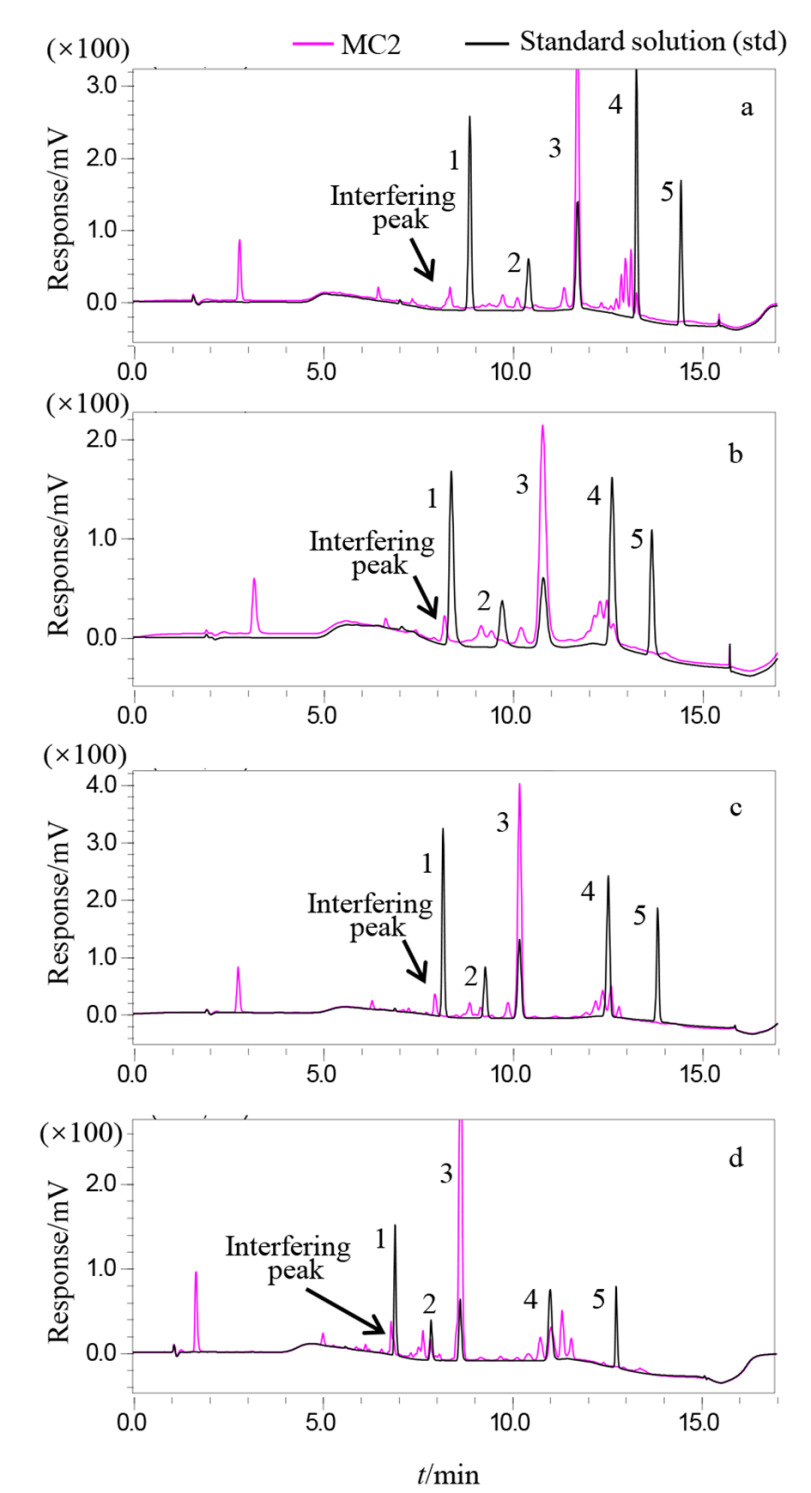
5种光引发剂混合标准溶液和MC2样品溶液经不同色谱柱分离的液相色谱图

**表1:** MC2样品加标溶液中5种光引发剂在不同色谱柱上的理论塔板数

Chromatographic column	Theoretical plate numbers				
	HCHPK	TPO-L	TPO	TMO	PB-TMBPO
Kromasil 100-5-C18	49857	44695	70928	184072	180464
Venusil MP C18	31796	24940	21921	56738	98669
Diamonsil plus C18	59293	49315	46112	96739	225576
Poroshell 120 EC-C18	68618	65329	55398	54890	248039

#### 2.1.4 温和梯度的优化

在选定的色谱条件下，考察了柱温分别为25、30、35和40 ℃时5种PIs与样品干扰峰的分离效果。结果显示，5种目标化合物在各柱温下彼此分离度均大于4。随着柱温降低，HCHPK与样品干扰物的分离度增大，但峰形会有展宽。当柱温为25 ℃时，色谱峰较宽；而当柱温为30 ℃时，色谱峰窄且HCHPK与样品干扰物分离度大于3，满足定量分析需求。在此基础上，进一步优化洗脱梯度，将梯度洗脱时间从17 min缩短到12 min。优化后的色谱条件在保持良好分离效果的同时，显著提高了分析效率。因此，最终确定的色谱条件为柱温30 ℃，梯度洗脱时间为12 min。

### 2.2 前处理条件的优化

#### 2.2.1 提取溶剂的选择

在甲油胶中PIs的检测中，样品前处理是确保复杂基质检测准确性和可靠性的关键环节。本研究选取了5种具有代表性的甲油胶（底胶、封层胶、纯色胶、亮片胶和渐变胶），系统评估了甲醇、乙腈、乙醇和丙酮4种溶剂对5种PIs的提取效率及对甲油胶样品分散性的影响。

实验结果表明，在超声功率500 W、时间20 min、室温条件下，4种溶剂对目标化合物的提取回收率均超过90%。尽管丙酮在分散性方面表现最佳，但其属于第3类易燃液体且为第3类易制毒化学品，受到公安部门严格管控，使用受限。甲醇和乙醇在处理有色甲油胶时分散性较差，存在明显的结块和黏附现象。相比之下，乙腈在初始分散溶解性方面优于甲醇和乙醇，略差于丙酮，但通过超声可达到与丙酮同样的提取率和分散度。综合考虑提取效率、溶剂安全性和操作便利性，本实验最终选择乙腈作为提取溶剂。

#### 2.2.2 取样量的选择

经检测，底胶、封层胶、亮片胶、纯色胶和渐变胶等甲油胶都含有目标PIs，且底胶和封层胶可完全溶解于乙腈。因此，从纯色胶、亮片胶和渐变胶中各选一批样品（G1、MN2、YH2），优先选择着色剂多、PIs多、黏度大的样品做取样量考察验证。3批样品分别称取0.05、0.10和0.15 g，采用10 mL乙腈和丙酮进行提取。以丙酮提取所得含量最大值为基准计算相对含量（%）；鉴于所有样品均未检出PB-TMBPO，向3批样品各加入0.40% PB-TMBPO，计算加标回收率。结果显示，5种PIs经2种溶剂提取的含量相对平均偏差范围为0.1%~3.3%，表明均提取完全；采用乙腈时的重复性（RSD为0.3%~1.0%）略优于丙酮（RSD为0.6%~2.1%）。此外，当取样量≥0.15 g时，PIs含量>4.0%的样品会超出线性范围；取样量为0.05 g时，需使用十万分之一天平。综合考虑，最终确定取样量为0.10 g。

#### 2.2.3 稳定性的考察

光照稳定性对PIs的检测准确性具有显著影响。本研究进一步考察了日光、灯光、避光以及室温和冷藏条件对目标PIs稳定性的影响。结果显示，5种PIs在避光和冷藏条件下贮存90天稳定；日光照射12 h，HCHPK剩余率为76.9%，TPO-L剩余率为4.2%，TPO、TMO、PB-TMBPO均完全降解；而实验室环境灯光照射24 h，5种PIs剩余率为98.9%~100.7%。这表明阳光对PIs稳定性的影响尤为显著。因此，为确保样品稳定性，所有操作应避光进行，样品提取液需在4℃冷藏保存，以最大限度降低光照对目标化合物的影响。

### 2.3 方法验证

#### 2.3.1 方法特异性

选择2批黏稠液体（MC1和MC2）、2批凝胶（MN1和YH1）以及含最多PIs的样品（MN2），按照前处理方法处理后进样测定，考察方法特异性。在优化的色谱条件下，记录试剂空白溶液、5个典型样品溶液、混合标准溶液和5个样品加标溶液的色谱图（见图3）。结果表明，在243 nm下，试剂空白无干扰峰，MC1、MN1、MN2均含有HCHPK的特征峰，该峰与基质中其他干扰峰的分离度均>2。在375 nm下，试剂空白无干扰峰，MC2、MC1、MN1、MN2均含有TPO的特征峰（峰3），YH1含有TPO-L的特征峰（峰2），MN2含有TMO的特征峰（峰4），这些PIs峰与基质中其他干扰峰分离度均良好，*R*>4，如图3b所示；在样品加标溶液中，5种PIs的色谱峰峰形良好，理论板数均不低于25 000，如图3c、3d所示。结果表明所建立的HPLC-DAD方法具有良好的方法特异性。

**图3:**
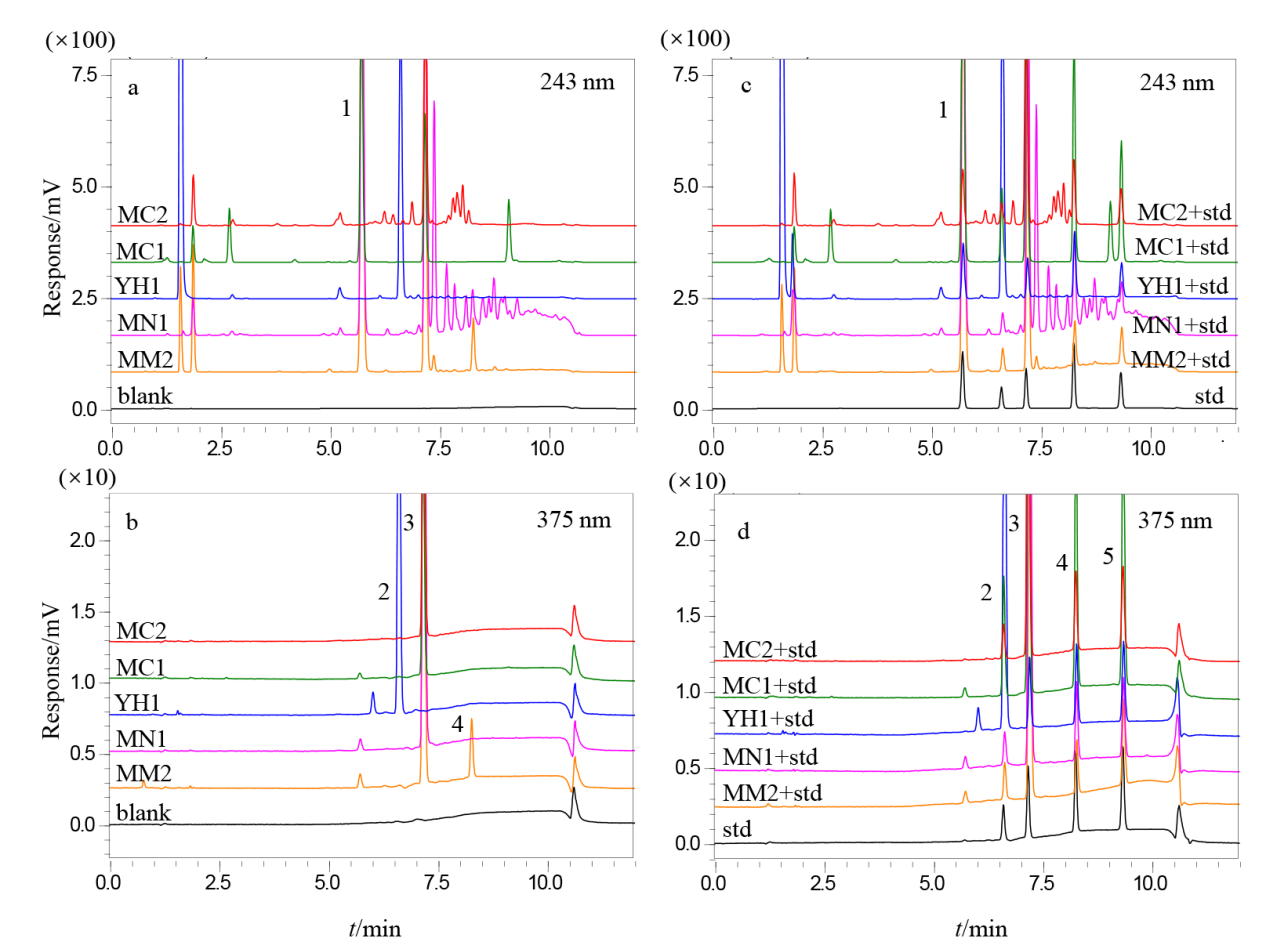
5个样品溶液和1个试剂空白溶液的（a）243 nm和（b）375 nm检测色谱图；5个样品加标溶液和1个混合标准溶液的（c） 243 nm和（d）375 nm检测色谱图

#### 2.3.2 线性关系、检出限与定量限

按1.2节配制不同质量浓度的系列混合标准溶液，按1.4节色谱条件进行测定，以各目标化合物的质量浓度为横坐标（*x*，mg/L）、峰面积为纵坐标（*y*）绘制标准曲线，5种目标化合物在2~600 mg/L范围内线性相关性良好，相关系数（*r*）均≥0.999 9。分别以3倍信噪比（*S/N*=3）和*S/N*=10所对应的质量浓度作为检出限（LOD）和定量限（LOQ），5种PIs的方法LOD为3.6~45 μg/g，方法LOQ为15~141 μg/g，结果见表2。

**表2:** 5种光引发剂的线性范围、线性方程、相关系数、检出限及定量限

Compound	Linear range/（mg/L）	Linear equation	*r*	LOD/（mg/L）	Method LOD/（μg/g）	LOQ/（mg/L）	Method LOQ/（μg/g）
HCHPK	1.95-586	*y*=1.39×10^4^ *x*-1.66×10^2^	0.9999	0.036	3.6	0.15	15
TPO-L	1.96-588	*y*=2.29×10^2^ *x*+5.52×10^2^	0.9999	0.45	45	1.41	141
TPO	1.94-582	*y*=4.78×10^2^ *x*+9.98	0.9999	0.38	38	0.87	87
TMO	1.96-587	*y*=5.23×10^2^ *x*+1.92×10^2^	0.9999	0.25	25	0.65	65
PB-TMBPO	1.96-588	*y*=6.06×10^2^ *x*+1.33×10^2^	0.9999	0.26	26	0.73	73

*y*： peak area； *x*： mass concentration， mg/L.

#### 2.3.3 回收率和精密度

甲油胶剂型主要分为液体/黏稠液体和凝胶两类。为验证所建方法的准确性和重复性，针对这两类代表性基质分别设计加标回收试验。虽然未购买到完全不含5种目标PIs的甲油胶样品，但不同样品目标物检出/未检出情况存在交叉，故每种剂型选取2批进行回收率验证。液体/黏稠液体：由于该类基质均检出TPO，因此向一批不含HCHPK、TPO-L、TMO和PB-TMBPO的甲油胶样品（MC2）中添加混合标准储备液，在0.4、4、40 mg/g 3个水平下进行HCHPK、TPO-L、TMO和PB-TMBPO的加标回收试验；向TPO本底含量为14.2 mg/g的甲油胶样品（MC1）中，添加混合标准储备液，在6、14、30 mg/g 3个加标水平下进行TPO加标回收试验。凝胶：向检出TPO-L但不含另4种PIs的甲油胶样品（YH1）中，添加混合标准储备液，在0.4、4、40 mg/g 3个水平下进行HCHPK、TPO、TMO和PB-TMBPO的加标回收试验；向另一批不含TPO-L的甲油胶样品（MN1）中，添加混合标准储备液，在0.4、4、40 mg/g 3个水平下进行TPO-L的加标回收试验。每种剂型每个浓度水平平行制备6份，按照1.3节提取后测定，计算5种PIs的回收率及相对标准偏差（RSD），结果见表3。在2类甲油胶基质中，各目标化合物的平均加标回收率为91.6%~101.8%，RSDs（*n*=6）为0.2%~4.3%。

**表3:** 5种光引发剂在2种甲油胶基质中的加标回收率与相对标准偏差（*n*=6）

Compound	Liquid/viscous liquid				Gel		
	Spiked/（mg/g）	Rec./ %	RSD/%		Spiked/（mg/g）	Rec./ %	RSD/%
HCHPK	0.4	91.6	0.3		0.4	97.6	0.2
	4	98.1	0.3		4	98.7	0.6
	40	99.0	0.4		40	99.7	0.3
TPO-L	0.4	97.4	4.3		0.4	99.4	3.3
	4	99.5	2.8		4	99.2	1.3
	40	99.7	0.4		40	99.6	0.3
TPO	6	100.4	3.1		0.4	99.3	3.9
	14	98.3	2.1		4	101.8	2.8
	30	100.1	1.3		40	99.6	0.3
TMO	0.4	96.7	3.0		0.4	99.8	3.1
	4	100.4	1.1		4	101.4	1.5
	40	99.5	0.4		40	99.4	0.3
PB-TMBPO	0.4	96.7	2.2		0.4	101.2	1.4
	4	99.2	0.4		4	99.2	0.9
	40	99.4	0.5		40	98.9	0.3

Rec.： recovery.

### 2.4 实际样品的检测

采用所建立的方法，对市售30批甲油胶样品进行检测（见图4）。检测过程中，若样品中检出的色谱峰保留时间与标准溶液一致，则进一步将样品的紫外光谱图与浓度相近的标准溶液光谱图进行比对，以确定待测化合物的检出情况。所有样品（包括底胶、封层胶和各类色胶）均检出1~3种PIs，总含量达产品的2.95%~9.66%（质量分数）；共检出4种PIs，每批均含有酰基氧化膦类PIs；有27批甲油胶检出欧盟禁用的TPO，4批样品检出TPO替代品TPO-L和TMO，而PB-TMBPO均未检出；TPO、TPO-L和TMO含量范围为1.42%~6.43%（质量分数）、6.73%~7.17%（质量分数）和0.25%（质量分数）；有23批检出*α*-羟基酮类PIs（HCHPK），含量为0.83%~4.84%（质量分数）。核对产品标签和备案资料发现，这些甲油胶均未标识含有检出的PIs成分。值得注意的是，其中5批样品检出的TPO含量已超出此前欧盟化妆品法规（EC）No 1223/2009附录Ⅲ第311项规定的最大允许使用含量5.0%（质量分数）^［12］^；而TPO也已于2025年9月1日起从欧盟化妆品限用物质清单移除，收录至禁用物质清单监管。因此，对甲油胶类化妆品中作为核心原料加入的TPO等PIs的检测和监管已迫在眉睫。

**图4:**
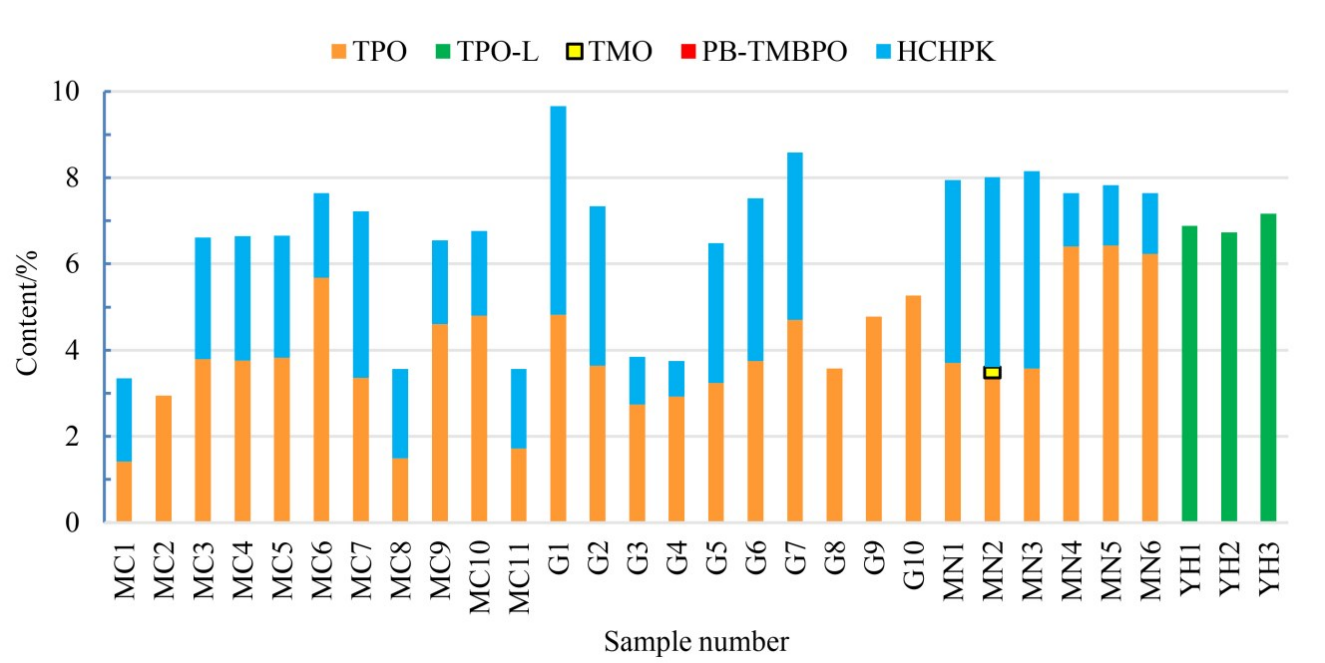
30批实际甲油胶样品的检测结果

## 3 结论

本研究开发了一种高效、可靠的HPLC-DAD方法，用于同时测定甲油胶中的5种关键PIs：TPO、TPO-L、TMO、HCHPK和PB-TMBPO。针对甲油胶复杂基质导致的检测干扰问题，本研究确立了双波长检测策略，并优化了流动相组成、色谱柱、前处理条件和稳定性。该方法成功克服了甲油胶中高含量色素和聚合物共洗脱的干扰，实现了5种PIs在常规C18柱上的分离检测。通过对甲油胶液体/黏稠液体和凝胶两种剂型的回收率和精密度验证，进一步证实了方法的准确性和可靠性，为基层实验室和产业界提供了一种低成本、易推广的实用工具。

本方法将TPO及其潜在替代物（TMO、TPO-L、PB-TMBPO）与目前产品常用的HCHPK纳入同一分析方法，以应对欧盟TPO禁令对我国化妆品监管和合规性评估带来的挑战。市售样品检测结果表明，该方法在市场监管、企业自控和产品迭代更新中具有即时应用价值。未来研究可基于本方法的实际检测数据，开展甲油胶中PIs的风险评估与限量标准制定，为产品质量提升和PIs绿色替代品开发提供支撑，满足法规和行业发展需求。
